# Biomarker-Guided Anti-EGFR Rechallenge Therapy in Metastatic Colorectal Cancer

**DOI:** 10.3390/cancers13081941

**Published:** 2021-04-17

**Authors:** Davide Ciardiello, Giulia Martini, Vincenzo Famiglietti, Stefania Napolitano, Vincenzo De Falco, Teresa Troiani, Tiziana Pia Latiano, Javier Ros, Elena Elez Fernandez, Pietro Paolo Vitiello, Evaristo Maiello, Fortunato Ciardiello, Erika Martinelli

**Affiliations:** 1 Oncologia Medica, Dipartimento di Medicina di Precisione, Università degli Studi della Campania Luigi Vanvitelli, 80131 Naples, Italy; davideciardiello@yahoo.it (D.C.); giulia.martini@unicampania.it (G.M.); vincenzo.famiglietti@yahoo.it (V.F.); stefania.napolitano@unicampania.it (S.N.); vincenzodefalc@gmail.com (V.D.F.); teresa.troiani@unicampania.it (T.T.); fjros@vhio.net (J.R.); fortunatociardiello@yahoo.com (F.C.); 2 Oncologia Medica, IRCCS Foundation Ospedale Casa Sollievo della Sofferenza, 71013 San Giovanni Rotondo, Italy; latianotiziana@gmail.com (T.P.L.); e.maiello@libero.it (E.M.); 3 Department of Medical Oncology, Vall d’Hebron University Hospital (HUVH), Vall d’Hebron Institute of Oncology (VHIO), IOB-Quiron, UVic-UCC, 08035 Barcelona, Spain; meelez@vhio.net; 4 Dipartimento di Oncologia, Istituto per la Ricerca sul Cancro (IRCC), Università di Torino, 10060 Candiolo, Italy; pietropaolo.vitiello@gmail.com

**Keywords:** rechallenge, anti-EGFR monoclonal antibodies, metastatic colorectal cancer

## Abstract

**Simple Summary:**

The survival of patients with metastatic colorectal cancer (mCRC) has been improved over the years and now reaches 30–40 months. However, few therapeutic options are available after failure of first- and second-line treatments. In fact, prognosis of chemo-refractory mCRC remains poor. Therefore, new therapeutic strategies are needed. Emerging evidence suggest that retreatment with epidermal growth factor (EGFR) inhibitors after a treatment break, in patients that obtained a clinical benefit by previous anti-EGFR, could lead to prolonged survival. The rationale beyond this “rechallenge” strategy is that after a “treatment holiday” EGFR resistant cancer cells decay, restoring the sensibility to EGFR blockade. In this review we analyze the current knowledge of retreatment with EGFR inhibitors, examine the role of novel biomarkers that can guide the appropriate selection of patients. Finally, we discuss future perspectives and on-going clinical trials.

**Abstract:**

The prognosis of patients with metastatic colorectal cancer (mCRC) who progressed to the first and the second lines of treatment is poor. Thus, new therapeutic strategies are needed. During the last years, emerging evidence suggests that retreatment with anti-epidermal growth factor receptor (EGFR) monoclonal antibodies (MAbs) in the third line of mCRC patients, that have previously obtained clinical benefit by first-line therapy with anti-EGFR MAbs plus chemotherapy, could lead to prolonged survival. The rationale beyond this “rechallenge” strategy is that, after disease progression to first line EGFR-based therapy, a treatment break from anti-EGFR drugs results in *RAS* mutant cancer cell decay, restoring the sensitivity of cancer cells to cetuximab and panitumumab. In fact, rechallenge treatment with anti-EGFR drugs has shown promising clinical activity, particularly in patients with plasma *RAS* and *BRAF* wild type circulating tumor DNA, as defined by liquid biopsy analysis at baseline treatment. The aim of this review is to analyze the current knowledge on rechallenge and to investigate the role of novel biomarkers that can guide the appropriate selection of patients that could benefit from this therapeutic strategy. Finally, we discuss on-going trials and future perspectives.

## 1. Background

Survival of patients with metastatic colorectal cancer (mCRC) has significantly improved over the past decades, mostly due to multimodal treatments, the optimization of chemotherapy regimens, and the use of targeted therapies and immunotherapy [1,2]. However, despite an increasing number of patients that are able to receive multiple lines of therapies, the overall clinical benefit is mainly due to first- and second-line treatments. In patients with untreated *RAS/BRAF* WT mCRC, the combination of a chemotherapy doublet (FOLFOX/FOLFIRI) with the epidermal growth factor (EGFR) blockade is associated with a mPFS of 12 months and a high response rate (RR) of approximately 60% [3,4,5]. After disease progression, second-line options are represented by the shift of the chemotherapy backbone and of monoclonal antibodies (MAbs) [1,2]. In this condition, combination of FOLFOX plus bevacizumab demonstrated a median PFS (mPFS) of 7 months and an RR of 22% [6]. Similar results were observed in the VELOUR trial, which investigated the addition of aflibercept to the FOLFIRI regimen as second-line treatment [7].

After failure of first- and second-line treatments, the standard of care (SOC) is represented by regorafenib (a small molecule multi-kinase inhibitor) or by trifluridine/tipiracil (an antimetabolite) [1,2,8,9,10]. Both drugs demonstrated a clinical activity in this chemorefractory setting with a mPFS of 2 months and a median OS (mOS) of 6–7 months [8,9,10]. However, major clinical responses to regorafenib and to trifluridine/tipiracil are rare (approximately 1%) with mostly disease stabilization. Therefore, new therapeutic options represent an unmet clinical need.

Recently, few studies have demonstrated that retreatment with anti-EGFR MAbs in the third or further lines of treatment in mCRC patients, which have previously obtained clinical benefit by first-line therapy with anti-EGFR MAbs plus chemotherapy, could lead to improved OS [11,12,13,14,15]. The rationale beyond this “rechallenge” strategy is that, after disease progression to first line EGFR-based chemotherapy, a treatment “holiday” from anti-EGFR drugs results in RAS mutant cancer cell decay, restoring the sensitivity of cancer cells to cetuximab or panitumumab [16,17,18]. Despite these promising results, in the absence of randomized phase III trials, there are still several open questions, including the optimal rechallenge therapy (cetuximab/panitumumab as single agents, combination with chemotherapy and/or immunotherapy), the appropriate selection of patients, and the identification of biomarkers of activity and efficacy.

The aim of this review is to analyze the current knowledge of rechallenge treatment, to examine the different treatment options and to investigate the role of novel biomarkers, that can guide patient selection. Finally, we will discuss ongoing clinical trials.

## 2. Rationale of Rechallenge Therapy

**Figure 1:**
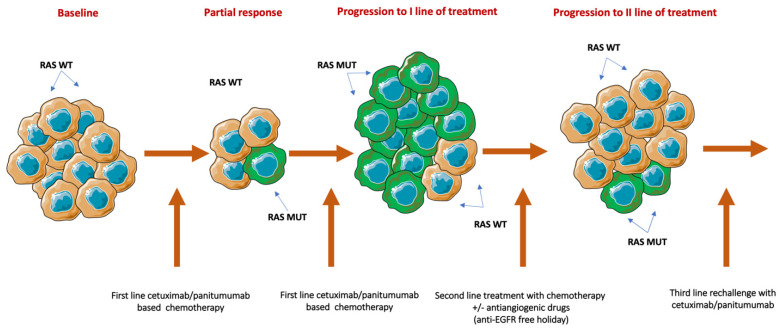
Biological rationale for rechallenge therapy. Treatment with anti-EGFR inhibitors rapidly eliminates *RAS* WT-sensitive clones and favors the expiation of resistant cancer cells. After disease progression, and due to the administration of a second line of chemotherapy without anti-EGFR monoclonal antibodies, RAS mutant clones progressively decay, inducing the proliferation of *RAS* WT cell. WT: Wild type; MUT: Mutant; /: Or.

Despite an initial response, the efficacy of anti-EGFR therapies can be limited by the presence of acquired mechanism(s) of cancer cell resistance. Approximately 30–40% of patients with baseline *RAS* wild type (WT) tumors, after treatment with cetuximab or panitumumab, displayed a mutation in *KRAS* or *NRAS* [19,20,21]. In fact, under the selective pressure of anti-EGFR MAbs, either a preexisting population of subclones carrying the resistance mutations can rapidly expand or de novo molecular resistance mutations can be developed, thus determining tumor progression [22,23,24]. In contrast, a subsequent treatment with chemotherapy plus bevacizumab can inhibit the proliferation of anti-EGFR-resistant clones, causing reduction or disappearance of *RAS* mutant subclones, potentially restoring anti-EGFR therapy sensibility [25]. In this scenario, the molecular landscape of CRC clones is continuously changing over time, due to the selection of different therapies (Figure 1). Morelli and colleagues showed, by analyzing serial circulating tumor DNA (ctDNA) samples of 62 RAS WT mCRC patients whose tumors developed resistance to EGFR inhibition (EGFRi), that the KRAS and EGFR mutant allele fraction (MAF) was inversely correlated with the time since last administration of anti-EGFR MAbs [21]. Moreover, Parseghian and colleagues found that, after the discontinuation of cetuximab and panitumumab, *KRAS* and *EGFR* mutant clones exponentially decay with half-life of 4.4 months [16]. These principles of clonal selection, associated with dynamic change in the cancer cell population, similarly apply also to other mechanisms of resistance to EGFRi [17,26,27]. Thus, after an anti-EGFR free therapeutic window, which resulted in the decrease of resistant clones, there is a biological rationale for retreatment with cetuximab or panitumumab.

## 3. Current Knowledge on Rechallenge Strategies and Possible Biomarkers

In the last years, an increasing number of studies investigated the possible role of anti-EGFR retreatment in the continuum of care of patients with *RAS* WT mCRC [23]. Different strategies were evaluated, including cetuximab or panitumumab as single agents, or coupled with chemotherapy (mostly with irinotecan) or with immunotherapy (Table 1). The first prospective trial was conducted by Santini and colleagues in 39 patients with heavily pretreated KRAS WT mCRC [11]. All patients had obtained a clinical benefit from previous cetuximab-based therapies, such as partial response (PR), complete response (CR), or stable disease (SD) for more than 6 months, then received at least a subsequent line of treatment (median four lines of therapy) and, finally were treated with irinotecan plus cetuximab as rechallenge strategy. The rationale behind this sequence of treatments was the evidence that cetuximab could restore the sensibility to irinotecan in chemorefractory patients [27,28,29,30,31]. Interestingly, the overall response rate (ORR) was 53.8%, with 19 PR (48.7%) and 2 CR (5.1%). Moreover, 14 patients (36%) experienced SD as best response. The median progression-free survival (mPFS) was 6.6 months (95%CI, 4.1–9.1 months). Almost 50% of the patients developed grade 3 drug-related adverse events (ADR). The most frequent adverse event was skin rash, with a significant correlation between skin toxicities during rechallenge and first cetuximab therapies (*p* = 0.01). These results are remarkable if we consider that mPFS with regorafenib or with trifluridine/tipiracil is approximately 2 months and that clinical responses are rare (1–2%) [8,9,10]. Unfortunately, these promising data were not confirmed by subsequent studies such as CRICKET and JACCRO CC-08, as discussed below [12,14]. One possible explanation is that the main cause of anti-EGFR first-line treatment interruption (disease progression vs. other causes, including treatment holyday in responding patients) was not reported. Therefore, it is difficult to establish the percentage of patients that received cetuximab plus irinotecan as true rechallenge strategy rather than as reintroduction of drugs to which the patient could be still responding.

The CRICKET trial was a small phase II proof of concept study that investigated rechallenge with cetuximab plus irinotecan as third-line treatment [12]. The main inclusion criteria were more restrictive as compared with the study of Santini et al. [11]. In particular, all patients had *RAS* and *BRAF* WT tumors received a first line with anti-EGFR MAbs plus irinotecan-based chemotherapy (FOLFIRI or FOLFOXIRI), obtained at least a PR with a mPFS of more than 6 months, progressed to a second line of chemotherapy containing oxaliplatin and bevacizumab, and had a EGFRi-free interval of a minimum of 4 months. In the intention to treat (ITT) the patient population, six out of 28 patients experienced PR (21%) with mPFS of 3.4 months (95% CI, 1.8–3.8 months) and with mOS of 9.8 months (95% CI, 5.2–13.1 months). The authors conducted a retrospective analysis on plasma samples to assess the potential predictive role of RAS mutations in ctDNA. Interestingly, no RAS mutation was detected at baseline in samples of patients that achieved PR. Moreover, mPFS was significantly higher in 13 patients with baseline RAS WT ctDNA as compared to 12 patients with baseline RAS mutant ctDNA (4.0 vs. 1.9 months; hazard ratio, HR, 0.44; 95% CI, 0.18–098; *p* = 0.03). A trend towards improved OS was reported (12.5 vs. 5.2 months; HR, 0.58; 95% CI, 0.22–1.52; *p* = 0.24). Despite the limitation of a small single-arm study, this is the first evidence that RAS WT status, as assessed by liquid biopsy, could represent an appropriate tool to select patients that might benefit of anti-EGFR rechallenge therapies [12].

The predictive role of liquid biopsy has been confirmed by two independent groups [13,15]. Sunakawa and colleagues reported a post hoc analysis of JACCRO CC-08 (*n* = 34) and CC-09 (*n* = 25) studies, which assessed rechallenge with irinotecan plus anti-EGFR mAbs for *KRAS* WT mCRC patients [13,14]. In 16 out of 59 patients, serial plasma samples were available for ctDNA analysis. No response was observed, with a disease control rate (DCR) of 62.5%. mPFS and mOS were of 3.1 and 8.9 months, respectively. At baseline evaluation, RAS mutations were found in 6/16 cases. Note that DCR was lower in KRAS mutant patients (33 vs. 80%), as well as mPFS (2.3 vs. 4.7 months; *p* = 0.016) and mOS (3.8 vs. 16 months; *p* = 0.0028). Interestingly, after progression, a total of 6/10 patients displayed *KRAS* or *NRAS* mutations. Moreover, post-progression survival was lower in patients that developed a *RAS* mutation compared with those that maintained a *RAS* WT status (4.8 vs. 7.2 months).

Recently, our group has reported the initial results of the CAVE mCRC trial, which investigated the combination of the anti-PD-L1mAb avelumab with cetuximab as an immune-rechallenge therapy in 77 patients with refractory mCRC [15]. Note that cetuximab (i) enhances NK cell-mediated antibody-dependent cell cytotoxicity (ADCC), (ii) favors the opsonization of cancer cells by dendritic cells, and (iii) increases Major Histocompatibility Complex (MHC) class II molecule expression with the recruitment of T cells in tumor microenvironment [32,33,34,35,36,37]. The main inclusion criteria were similar to the CRICKET study [12]. The trial met the primary endpoint, reaching an mOS of 13.1 months (95% CI, 7.2–18.9 months). mPFS was 3.6 months (95% CI, 3.2–4.1 months). Six out of 77 patients obtained CR (1, 1.8%) or PR (5, 6%), whereas 44 patients (57%) experienced SD as best response. Plasma samples from 56 patients were suitable for liquid biopsy analysis. *KRAS, NRAS*, and *BRAF* ctDNA were WT in 48 patients, while 19 patients displayed a mutation in *RAS* and/or *BRAF* ctDNA at baseline. Note that patients with *RAS/BRAF* WT ctDNA had mOS of 16.3 months (95% CI, 9.0–24.1 months) compared to 11.5 months (95% CI, 5.4–17.5 months) in patients with mutated ctDNA. mPFS was 4.3 months (95% CI, 3.0–5.5 months) in *RAS/BRAF* WT patients compared to 3.0 months (95% CI, 2.6–3.3 months) in mutated patients. Treatment was well tolerated. The most frequent grade 3 adverse events were skin rash (14%) and diarrhoea (4%).

So far, liquid biopsy might represent a promising, noninvasive tool to select patients with *RAS/BRAF* WT tumors that are amenable to receive an anti-EGFR rechallenge therapy. Unfortunately, liquid biopsy is not approved or routinely available in all countries. Therefore, additional predictive biomarkers are needed for patient selection.

The biological motivation of EGFRi reintroduction is correlated with the clonal decay of resistant cell, after an anti-EGFR free therapeutic window [16]. In a large retrospective study by Liu X and colleagues, which included 89 patients that received cetuximab ± erlotinib, a longer interval between the two anti-EGFR based therapy was correlated with a clinical benefit (*p* = 0.053) [38]. Moreover, the mPFS of rechallenge therapy was longer in patients that responded to prior therapy with cetuximab/panitumumab (4.9 vs. 2.5 months). Similar results were observed in the JACCRO CC-08 study, in which 34 patients with *KRAS* WT mCRC received irinotecan and cetuximab as third line rechallenge treatment [14]. In the ITT population, mPFS and mOS were, respectively, 2.4 and 8.2 months. ORR and DCR were 2.9% and 55.9%, respectively. A *post hoc* analysis was conducted to evaluate additional biomarkers. Interestingly, a longer cetuximab-free interval (>372 days) was correlated with improved mPFS (4.6 vs. 2.1 months; HR, 0.31; 95%CI, 0.18–0.86; *p* = 0.020) and mOS (14.1 vs. 6.3 months; HR0.31; 95% CI, 0.13–0.74, *p* = 0.008).

In a retrospective study, 14 patients with chemorefractory mCRC received irinotecan plus cetuximab as retreatment strategy [39]. The ORR was 21.4% (3/14) and SD was 50% (7/14), with mPFS of 4.4 months (95% CI, 1.4–5.6). A significant correlation between mPFS after rechallenge therapy and the EGFRi-free interval was observed (r = 0.08; *p* = 0.79). Furthermore, patients exposed to a longer duration of first cetuximab-based regimen exhibited better outcomes.

Recently, Rossini and colleagues reported the results of a retrospective analysis of 86 patients with refractory mCRC that received retreatment with anti-EGFR MAbs [40]. The ORR was 19.8%, and mPFS and mOS were 3.8 and 10.2 months, respectively. The authors investigated several clinical variables to identify possible biomarkers to predict the response to anti-EGFR rechallenge. In contrast with previous findings, longer mPFS and mOS were not affected by the time from the last administration of cetuximab or by the cause for discontinuation of first-line treatment. Note that a higher ORR was observed in patients with longer anti-EGFR free interval (>15 months) and that received more than 2 prior line of treatments.

Recently, two small retrospective studies, including 17 and 22 patients, which evaluated the role of retreatment with anti-EGFR MAbs, have been published. Despite a promising mPFS of approximately 4 months as compared to historical controls, these findings did not translate in improved overall survival [41,42,43].

**Table 1:** Completed rechallenge studies.

Study	Study Type	Number of Patients	Rechallenge Treatment	RR	mPFS	mOS
Santini et al., 2012 [11]	Retrospective	39	FOLFIRI + Cetuximab Irinotecan + Cetuximab	53.8%	6.6 m	NR
CRICKET	Prospective	28	Irinotecan + Cetuximab	21.4%	3.4 m	9.8
CRICKET (*RAS* ctDNA WT)	Prospective	13	Irinotecan + Cetuximab	31%	4 m	12.5 m
CRICKET (*RAS* ctDNA MUT)	Prospective	12	Irinotecan + Cetuximab	0%	1.9 m	5.2 m
Sunakawa Y et al., 2020 [13]	Prospective	16	Irinotecan + anti-EGFR	0%	3.1 m	8.9 m
Sunakawa Y et al., 2020 (*RAS* ctDNA WT) [13]	Prospective	10	Irinotecan + anti-EGFR	0%	4.7 m	16 m
Sunakawa Y et al., 2020 (*RAS* ctDNA MUT) [13]	Prospective	6	Irinotecan + anti-EGFR	0%	2.3 m	3.8 m
CAVE	Prospective	77	Cetuximab + Avelumab	7.8%	3.6 m	13.1 m
CAVE (*RAS/BRAF/* *EGFR* ctDNA WT)	Prospective	48	Cetuximab + Avelumab	8.5%	4.3 m	16.3 m
CAVE (*RAS/BRAF/* *EGFR* ctDNA MUT)	Prospective	19	Cetuximab + Avelumab	5.1%	3 m	11.5 m
JACCRO CC-08	Prospective	34	Irinotecan + Cetuximab	0%	2.4 m	8.1 m
Liu X et al., 2015 [38]	Retrospective	89	Cetuximab ± Erlotinib	NR	4.9 m (prior responder) 2.5 m (no responder)	NR
Tanioka H et al., 2018 [39]	Retrospective	14	Irinotecan + Cetuximab	21.4%	4.4 m	NR
Rossini D et al., 2020 [40]	Retrospective	86	Panitumumab/Cetuximab/FOLFIRI + Cetuximab/ FOLFOX + Panitumumab/CapIRI + Cetuximab/Irinotecan + Panitumumab/ Irinotecan + Cetuximab	19.8%	3.8 m	10.2 m
Karani A et al., 2020 [42]	Retrospective	17	Cetuximab ± CT	18%	3.3 m	8.4 m
Chong L et al. 2020 [43]	Retrospective	22	Cetuximab/Panitumumab	4.5%	4.1 m	7.7 m

RR: Response rate; mPFS: median progression free survival; mOS: median overall survival; m: Months; NR: Not reported; ctDNA: circulating tumor DNA; WT: Wild type; MUT: Mutant; EGFR: Epidermal growth factor receptor; CT: Chemotherapy.

## 4. Discussion

The mOS of patients with *RAS* WT mCRC have improved over time and now it reaches 30 to 40 months [1,2]. In fact, an increasing number of patients maintain a good performance status after progression to first-line chemotherapy regimens in combination with anti-EGFR or with antiangiogenic MAbs, and therefore they are suitable for further lines of treatment [44]. In this setting, treatment goals are represented by prolongation of survival, improvement in symptom control and preservation of good quality of life. Regorafenib and trifluridine/tipiracil represent the standard of care (SOC) in patients that progressed to first- and second-line therapies [1,2,3,4,5]. Both drugs showed a small, yet significant, clinical activity compared to placebo, reporting a mOS and mPFS of approximately 7 and 2 months, respectively. However, this limited efficacy is associated with a relevant toxicity. The safety profile of regorafenib is challenging, with 50% of patients experiencing grade 3 ADR, including hand-foot syndrome, rash, fatigue, diarrhea, and hypertension [3]. On the other hand, trifluridine/tipiracil displays a remarkable hematologic toxicity that could be treatment limiting in heavily pretreated patients.

Different retrospective studies have evaluated the safety and clinical efficacy of rechallenge with 5-FU/capecitabine plus oxaliplatin in pretreated patients with mCRC [45]. Despite the heterogeneity in study populations that could jeopardize the interpretation of the results, signals of activity have been observed. The most frequent grade 3 or 4 ADR were hematologic toxicities (5–27%), peripheral neuropathy (5–14%), and hypersensitivity reactions (5–20%). However, well-designed prospective trials are required to clarify the possible role of oxalipatin-based regimens retreatment.

In this scenario, rechallenge with anti-EGFR MAbs may represent a fascinating therapeutic option. However, in the absence of phase III randomized trials, there are several open questions, including the choice of the best rechallenge regimen, as well as the identification of predictive biomarkers of response to guide patient selection. The most used rechallenge regimen in prospective and retrospective trials is represented by the combination of irinotecan plus cetuximab [11,12,13,14,37,38,40,41,42]. The main advantage of this scheme is that both compounds are largely available in clinical practice. Moreover, the reintroduction of an active chemotherapy could determine tumor shrinkage, with ORR near to 20% in selected patients, which looks favorable as compared to regorafenib or trifluridine/tipiracil [8,9,10]. Nevertheless, this treatment could determine significant adverse events, such as grade 3 skin toxicity, diarrhea, and neutropenia. The impact of these toxicities on subsequent lines of treatments with regorafenib and trifluridine/tipiracil needs to be assessed. Further, a major limitation of these studies is represented by the absence of a control arm with SOC or with cetuximab/panitumumab as single agents.

The CAVE mCRC has introduced the novel concept of immune-rechallenge by combining cetuximab with the anti-PD-L1 MAb avelumab [15]. This treatment has demonstrated a potentially relevant clinical activity with significantly prolonged survival at the cost of acceptable toxicity. However, despite having a significant preclinical rationale, the real advantage of adding immune therapy to cetuximab has yet to be confirmed in a randomized trial.

Finally, several phase II studies are currently ongoing to evaluate different rechallenge strategies in mCRC (Table 2). VELO is a large, randomized phase II study that is including 112 patients with *RAS* WT mCRC, and it evaluates panitumumab plus trifluridine/tipiracil versus trifluridine/tipiracil as third-line therapy in patients that have obtained clinical benefit from first line anti-EGFR therapy (EudraCT Number 2018-001600-12). The primary endpoint is PFS and the secondary endpoints are ORR and OS. This trial is currently recruiting. PARERE is a randomized phase II study investigating the best retreatment sequence strategy between panitumumab followed by regorafenib, and vice versa, in patients with *RAS* and *BRAF* WT chemorefractory mCRC (EudraCT Number 2019-002834-35). The main inclusion criteria are similar with the CRICKET trial. However, *RAS* and *BRAF* WT ctDNA in the plasma is mandatory before study enrollment. The primary endpoint is OS and the secondary endpoints are PFS1, PFS2, and ORR. PULSE (NCT03992456) is a randomized phase II study, investigating rechallenge with panitumumab vs. SOC (regorafenib or trifluridine/tipiracil) in 120 patients with refractory *RAS* WT mCRC. The primary endpoint is OS. FIRE4 is a randomized phase III study including 550 patients with *RAS* WT mCRC to evaluate irinotecan plus cetuximab vs. regorafenib or another anti-EGFR free treatment as a third-line therapy in patients with *RAS* WT mCRC. These patients were treated with FOLFIRI plus cetuximab at as a first-line treatment (obtaining CR/PR with PFS >6 months) and after disease progression received FOLFOX plus bevacizumab as a second-line treatment (NCT02934529). The primary endpoint was OS from randomization to third-line treatment. In OS3, patients responded to treatment with cetuximab under a cetuximab rechallenge vs. an anti-EGFR-free treatment. Other ongoing clinical trials assessing cetuximab/panitumumab ± chemotherapy as rechallenge strategies are summarized in Table 2.

The main motivation of rechallenge is that, in the absence of direct pressure from anti-EGFR therapy, there is a progressive decay of resistant clones, restoring the sensibility to cetuximab/panitumumab [16]. In the study by Parseghian and colleagues, the median half-time of *RAS* mutant clone decay was 4 months [16]. The duration of the anti-EGFR treatment-free interval before rechallenge remains an unsolved question. In the CRICKET and CAVE mCRC trials, a period of at least 4 months after the discontinuation of EGFRi was required. Of note, *post hoc* subgroup analysis showed that a longer anti-EGFR-free interval was associated with better outcomes [14,38,39]. These data were not confirmed by the retrospective study by Rossini and colleagues [40].

The PROSPECT-C trial is a biomarker study to assess the mechanism of resistance in a cohort of 47 patients with chemorefractory *RAS WT* mCRC that received cetuximab as single agent [46]. A translational analysis was performed including tissue biopsy (when feasible) and liquid biopsy (every four weeks). The study validated the role of liquid biopsy in identifying mechanism(s) of acquired resistance, even before the insurgence of clinical or radiologic PD, and in line with previous studies, it confirmed that *RAS* mutant clones faded after anti-EGFR treatment discontinuation [11,16].

So far, pretreatment baseline *RAS/BRAF/EGFR* WT ctDNA in the plasma is the main potential predictive biomarker of response to anti-EGFR rechallenge [12,13,15]. Therefore, liquid biopsy could represent a noninvasive and highly sensitive tool for selecting patients. In this regard, different clinical trials are currently ongoing (Table 2). CHRONOS is a phase II study which is assessing the role of liquid biopsy for vertical monitoring of *RAS* status in 27 patients with baseline *RAS* WT mCRC (NCT03227926). In the “molecular screening phase”, patients with *RAS* WT mCRC will receive an anti-EGFR mAb-based therapy and upon progression a subsequent line of treatment. Plasma will be collected at baseline and after disease progression to first and to second line of treatment. Thus, patients showing a >50% drop in *RAS* mutational load at the time of rechallenge as compared to baseline mutational load after disease progression to first-line therapy will be eligible for the “trial phase”. The study will be considered positive if rechallenge with panitumumab achieved a response rate of 30% or more.

Emerging evidence suggests that a subset of patients with RAS/BRAF WT mCRC, after progression to first-line cetuximab-based chemotherapy, continue to take advantage of EGFRi [47,48,49]. The highest benefit has been observed in baseline *RAS* WT ctDNA tumors [50]. The CAPRI 2 GOIM is a large multicenter phase II sequence strategy study, which assesses the continuum of care in patients with *RAS/BRAF* WT mCRC (EudraCT2020-003008). Two-hundred patients will be treated with FOLFIRI plus cetuximab as first-line therapy. After progression, patients with *RAS/BRAF* WT tumor according to liquid biopsy will continue EGFR blockade while changing chemotherapy backbone (FOLFOX). In contrast, patients with *RAS* or *BRAF* ctDNA mutations will shift to FOLFOX plus bevacizumab. Finally, after a subsequent disease progression, patients with *RAS/BRAF* WT ctDNA will be treated with irinotecan plus cetuximab, whereas patients with *RAS* or *BRAF* mutations will receive regorafenib or trifluridine/tipiracil as SOC third-line therapy.

## 5. Conclusions

Retreatment with EGFRi represents a promising therapeutic strategy for patients with refractory mCRC. To date, based on the evidence of retrospective or small phase II clinical trials, the best candidates to receive EGFRi retreatments seem to be patients that obtained a CR/PR to first line anti-EGFR based chemotherapy. Although the optimal duration of the anti-EGFR-free window is still under evaluation, a treatment break of at least four months has been considered adequate, on the basis of pre-clinical and translational findings. So far, the only recognized predictive biomarker of response is the absence of baseline *RAS, BRAF,* and *EGFR* ctDNA mutations. Therefore, liquid biopsy may represent a promising tool for patient stratification and selection and should be considered as a key inclusion criterion for future clinical trials. The identification of novel biomarkers is an unmet clinical need, therefore further research is required.

The most used rechallenge strategy is represented by the combination of irinotecan plus cetuximab. However, the real advantage over retreatment with single agent cetuximab/panitumumab has yet to be determined. Similarly, the combination of cetuximab plus avelumab showed an interesting clinical activity, with a favorable safety profile. However, these results have to be confirmed by large, randomized trials comparing the immune-rechallenge with cetuximab and/or SOC.

## Figures and Tables

**Table 2:** Rechallenge with anti-epidermal growth factor ongoing trials.

Study Name	Phase	Number of Patient	Treatment Strategy	Liquid Biopsy Selection
VELO	II	112	Trifluridine/tipiracil + Panitumumab vs. Trifluridine/tipiracil	No
PARERE	II	220	Panitumumab > Regorafenib vs. Regorafenib > Panitumumab	Yes
PULSE	II	120	Panitumumab vs. Trifluridine/tipiracil or Regorafenib	Yes
FIRE-4	III	550	I line FOLFIRI + Cetuximab II line FOLFOX + Bevacizumab III Irinotecan + Cetuximab vs. Regorafenib or another anti-EGFR free treatment	No
A-REPEAT	II	33	FOLFIRI/FOLFOX + Panitumumab	No
NCT03524820	II	60	I line anti-EGFR + chemotherapy II line chemotherapy III line Cetuximab ± chemotherapy	No
CHRONOS	II	27	I line anti-EGFR + chemotherapy II line chemotherapy III line Panitumumab	Yes
CAPRI II GOIM	II	200	I line FOLFIRI + Cetuximab II Line FOLFOX + Cetuximab vs. FOLFOX + Bevacizumab III line Irinotecan + Cetuximab vs. Trifluridine/tipiracil or Regorafenib	Yes

EGFR, epidermal growth factor receptor; /:OR.
