# Correction: Ciardiello et al. Biomarker-Guided Anti-EGFR Rechallenge Therapy in Metastatic Colorectal Cancer. *Cancers* 2021, *13*, 1941

**DOI:** 10.3390/cancers14163900

**Published:** 2022-08-12

**Authors:** Davide Ciardiello, Giulia Martini, Vincenzo Famiglietti, Stefania Napolitano, Vincenzo De Falco, Teresa Troiani, Tiziana Pia Latiano, Javier Ros, Elena Elez Fernandez, Pietro Paolo Vitiello, Evaristo Maiello, Fortunato Ciardiello, Erika Martinelli

**Affiliations:** 1 Oncologia Medica, Dipartimento di Medicina di Precisione, Università degli Studi della Campania Luigi Vanvitelli, 80131 Naples, Italy; 2 Oncologia Medica, IRCCS Foundation Ospedale Casa Sollievo della Sofferenza, 71013 San Giovanni Rotondo, Italy; 3 Department of Medical Oncology, Vall d’Hebron University Hospital (HUVH), Vall d’Hebron Institute of Oncology (VHIO), IOB-Quiron, UVic-UCC, 08035 Barcelona, Spain; 4 Dipartimento di Oncologia, Istituto per la Ricerca sul Cancro (IRCC), Università di Torino, 10060 Candiolo, Italy

## Affiliation Correction

In the original publication [1], there was an error regarding the affiliation 2, the correct affiliation should be “Oncologia Medica, IRCCS Foundation Ospedale Casa Sollievo della Sofferenza, 71013 San Giovanni Rotondo, Italy”.

## Error in Table

There were some typographical errors in Table 1. The results of the CAVE mCRC (lines 9, 10 and 11 of Table 1) were not correctly reported. The corrected Table 1 appears below. 

**Table 1:** Completed rechallenge studies.

Study	Study Type	Number of Patients	Rechallenge Treatment	RR	mPFS	mOS
Santini et al., 2012 [11]	Retrospective	39	FOLFIRI + Cetuximab Irinotecan + Cetuximab	53.8%	6.6 m	NR
CRICKET	Prospective	28	Irinotecan + Cetuximab	21.4%	3.4 m	9.8
CRICKET (*RAS* ctDNA WT)	Prospective	13	Irinotecan + Cetuximab	31%	4 m	12.5 m
CRICKET (*RAS* ctDNA MUT)	Prospective	12	Irinotecan + Cetuximab	0%	1.9 m	5.2 m
Sunakawa Y et al., 2020 [13]	Prospective	16	Irinotecan + anti-EGFR	0%	3.1 m	8.9 m
Sunakawa Y et al., 2020 (*RAS* ctDNA WT) [13]	Prospective	10	Irinotecan + anti-EGFR	0%	4.7 m	16 m
Sunakawa Y et al., 2020 (*RAS* ctDNA MUT) [13]	Prospective	6	Irinotecan + anti-EGFR	0%	2.3 m	3.8 m
CAVE	Prospective	77	Cetuximab + Avelumab	7.8%	3.6 m	13.1 m
CAVE (*RAS/BRAF/* *EGFR* ctDNA WT)	Prospective	48	Cetuximab + Avelumab	8.5%	4.3 m	16.3 m
CAVE (*RAS/BRAF/* *EGFR* ctDNA MUT)	Prospective	19	Cetuximab + Avelumab	5.1%	3 m	11.5 m
JACCRO CC-08	Prospective	34	Irinotecan + Cetuximab	0%	2.4 m	8.1 m
Liu X et al., 2015 [38]	Retrospective	89	Cetuximab ± Erlotinib	NR	4.9 m (prior responder) 2.5 m (no responder)	NR
Tanioka H et al., 2018 [39]	Retrospective	14	Irinotecan + Cetuximab	21.4%	4.4 m	NR
Rossini D et al., 2020 [40]	Retrospective	86	Panitumumab/Cetuximab/FOLFIRI + Cetuximab/ FOLFOX + Panitumumab/CapIRI + Cetuximab/Irinotecan + Panitumumab/ Irinotecan + Cetuximab	19.8%	3.8 m	10.2 m
Karani A et al., 2020 [42]	Retrospective	17	Cetuximab ± CT	18%	3.3 m	8.4 m
Chong L et al., 2020 [43]	Retrospective	22	Cetuximab/Panitumumab	4.5%	4.1 m	7.7 m

RR: Response rate; mPFS: median progression free survival; mOS: median overall survival; m: Months; NR: Not reported; ctDNA: circulating tumor DNA; WT: Wild type; MUT: Mutant; EGFR: Epidermal growth factor receptor; CT: Chemotherapy.

In Table 2, information regarding the FIRE-4 (NCT02934529) clinical trial was not correctly reported. The corrected Table 2 appears below.

**Table 2:** Rechallenge with anti-epidermal growth factor ongoing trials.

Study Name	Phase	Number of Patient	Treatment Strategy	Liquid Biopsy Selection
VELO	II	112	Trifluridine/tipiracil + Panitumumab vs. Trifluridine/tipiracil	No
PARERE	II	220	Panitumumab > Regorafenib vs. Regorafenib > Panitumumab	Yes
PULSE	II	120	Panitumumab vs. Trifluridine/tipiracil or Regorafenib	Yes
FIRE-4	III	550	I line FOLFIRI + Cetuximab II line FOLFOX + Bevacizumab III Irinotecan + Cetuximab vs. Regorafenib or another anti-EGFR free treatment	No
A-REPEAT	II	33	FOLFIRI/FOLFOX + Panitumumab	No
NCT03524820	II	60	I line anti-EGFR + chemotherapy II line chemotherapy III line Cetuximab ± chemotherapy	No
CHRONOS	II	27	I line anti-EGFR + chemotherapy II line chemotherapy III line Panitumumab	Yes
CAPRI II GOIM	II	200	I line FOLFIRI + Cetuximab II Line FOLFOX + Cetuximab vs. FOLFOX + Bevacizumab III line Irinotecan + Cetuximab vs. Trifluridine/tipiracil or Regorafenib	Yes

EGFR, epidermal growth factor receptor; /:OR.

## Text Correction

1. There was a typing error in the original paper, all “Trifluoridine” should be changed to “Trifluridine”.

2. There was also an error regarding information about the FIRE4 clinical trial, specifically the number of patients included in the study and the primary endpoint. In the fourth paragraph on page 8, the original sentences read as follows: 

“FIRE4 is a randomized phase II study including 230 patients with *RAS* WT mCRC and has the aim to evaluate irinotecan plus cetuximab vs. SOC as third-line therapy in patients with *RAS* WT mCRC, that have been treated with FOLFIRI plus cetuximab at first line (obtaining CR/PR with PFS >6 months) and after disease progression have received FOLFOX plus bevacizumab as second line treatment. The primary endpoint is OS.” 

These should be changed to the following: 

“FIRE4 is a randomized phase III study including 550 patients with *RAS* WT mCRC to evaluate irinotecan plus cetuximab vs. regorafenib or another anti-EGFR free treatment as a third-line therapy in patients with *RAS* WT mCRC. These patients were treated with FOLFIRI plus cetuximab at as a first-line treatment (obtaining CR/PR with PFS >6 months) and after disease progression received FOLFOX plus bevacizumab as a second-line treatment (NCT02934529). The primary endpoint was OS from randomization to third-line treatment. In OS3, patients responded to treatment with cetuximab under a cetuximab rechallenge vs. an anti-EGFR-free treatment.” 

The authors apologize for any inconvenience caused and state that the scientific conclusions are unaffected. The original article has been updated.
